# Novel Associations Between *METTL3* Gene Polymorphisms and Pediatric Acute Lymphoblastic Leukemia: A Five-Center Case-Control Study

**DOI:** 10.3389/fonc.2021.635251

**Published:** 2021-09-09

**Authors:** Xiaoping Liu, Libin Huang, Ke Huang, Lihua Yang, Xu Yang, Ailing Luo, Mansi Cai, Xuedong Wu, Xiaodan Liu, Yaping Yan, Jianyun Wen, Yun Cai, Ling Xu, Hua Jiang

**Affiliations:** ^1^Department of Hematology, Guangzhou Women and Children’s Medical Center, Guangzhou Medical University, Guangzhou, China; ^2^Pediatrics Department, The First Affiliated Hospital, Sun Yat-Sen University, Guangzhou, China; ^3^Department of Pediatrics, Sun Yat-sen Memorial Hospital, Sun Yat-sen University, Guangzhou, China; ^4^Pediatric Center of Zhujiang Hospital, Southern Medical University, Guangzhou, China; ^5^Department of Pediatrics, Nanfang Hospital, Southern Medical University, Guangzhou, China; ^6^Division of Birth Cohort Study, Guangzhou Women and Children’s Medical Center, Guangzhou Medical University, Guangzhou, China; ^7^Department of Pediatrics, The Third Affiliated Hospital, Sun Yat-sen University, Guangzhou, China

**Keywords:** methyltransferase-like 3, acute lymphoid leukemia, polymorphism, pediatric, susceptibility

## Abstract

**Objective:**

To reveal the contributing role of METTL3 gene SNPs in pediatric ALL risk.

**Patients and Methods:**

A total of 808 pediatric ALL cases and 1,340 cancer-free controls from five hospitals in South China were recruited. A case-control study by genotyping three SNPs in the *METTL3* gene was conducted. Genomic DNA was abstracted from peripheral blood. Three SNPs (rs1263801 C>G, rs1139130 A>G, and rs1061027 A>C) in the METTL3 gene were chosen to be detected by taqman real-time polymerase chain reaction assay.

**Results:**

That rs1263801 C>G, rs1139130 A>G, and rs1061027 A>C polymorphisms were significantly associated with increased pediatric ALL risk was identified. In stratification analyses, it was discovered that rs1263801 CC, rs1061027 AA, and rs1139130 GG carriers were more likely to develop ALL in subgroups of common B-ALL, MLL gene fusion. Rs1263801 CC and rs10610257 AA carriers were more possible to increase the risk of ALL in subgroups of low hyperdiploid, and all of these three SNPs exhibited a trend toward the risk of ALL. All of these three polymorphisms were associated with the primitive/naïve lymphocytes and MRD in marrow after chemotherapy in ALL children. Rs1263801 CC and rs1139130 AA alleles provided a protective effect on MRD ≥0.01% among CCCG-treated children. As for rs1139130, AA alleles provided a protective effect on MRD in marrow ≥0.01% on 33 days and 12 weeks among CCCG-treated children, but provided a risk effect on MRD in the marrow ≥0.01% among SCCLG-treated children. As for rs1263801 CC and rs1139130 AA, these two alleles provided a protective effect on MRD in the marrow ≥0.01% among CCCG-treated children.

**Conclusion:**

In this study, we revealed that *METTL3* gene polymorphisms were associated with increased pediatric ALL risk and indicated that *METTL3* gene polymorphisms might be a potential biomarker for choosing ALL chemotherapeutics.

## Introduction

Acute lymphoblastic leukemia (ALL) is the most common type of pediatric cancer in the world; in China, it accounts for 70–80% of pediatric leukemia (1). ALL can be classified by immune cell phenotype as B-cell ALL and T-cell ALL. B-cell ALL is the most common ALL; T-cell ALL is typically more aggressive (2). As traditional chemotherapy combined with novel therapies makes great progress, higher survival rates and reduced morbidities have been achieved in ALL. Recently, the 5-year overall survival rate of ALL children younger than 14 years has been achieved >90% (3). However, recurrence occurs in 15–20% of ALL children, and 15% pediatric ALL patients were therapeutic failures, which resulted in early age mortality (4). ALL is characterized by multiple genetic alterations (5).

Heritable variations in genes are risk factors for ALL and play a strong role in the development of pediatric ALL (6). Populations with different races are well distinguished by genetic polymorphisms. Genome-wide association studies (GWAS) have identified a number of loci, and single nucleotide polymorphism (SNP) associations in several genes are associated with the risk of ALL. Genetic alterations in pediatric ALL are found to be very different from those in adult ALL (7).

N^6^-methyladenosine (m6A) is the most abundant internal modification of messenger RNAs (mRNAs) in eukaryotic organisms. Methylation at the sixth N atom on adenine base is m6A. M6A regulates mRNA expression posttranscriptionally in a dynamic and reversible manner (8). M6A modification is regulated by several key regulators, including writers [RNA methyltransferase complex methyltransferase-like 3 (METTL3)/methyltransferase-like 14 (METTL14)/Wilms’ tumor 1-associating protein (WTAP)], erasers [demethylases fat mass and obesity-associated protein (FTO) and AlkB homolog 5 (ALKBH5)], and readers (YTHD family proteins) (9). It was reported that dysregulation of m6A is associated with multiple tumors including acute myeloid leukemia (AML) (10). M6A methylation writer METTL3 was discovered playing an oncogenic role in carcinogenesis, such as colorectal carcinoma (11), bladder cancer (12), breast cancer (13), etc. METTL3 mRNA and protein are expressed abundantly in AML cells, and their depletion induces cell differentiation and apoptosis and delays leukemia progression (14). Some genetic variations in m6A-related gene regions may affect m6A methylation, subsequently regulating mRNA expression (15). Studies identified that m6A-associated SNPs were potential functional variants for periodontitis (16) and coronary artery disease (17). Genetic alterations in the m6A demethyltransferase FTO gene were shown to be associated with ALL and AML risk, and there is evidence that indicates dysregulation of m6A methyltransferase METTL3 in AML (18, 19). However the relationship between genetic variations of the METTL3 gene and ALL is still unclear.

In the present study, a total of three SNPs were selected to assess the relationship between *METTL3* polymorphisms and pediatric ALL. The current study was a case-control study that was performed using samples from five hospitals in South China.

## Materials and Methods

### Study Subjects

A Southern Chinese population that included 808 pediatric ALL patients and 1,340 age-matched, gender-matched, and ethnicity-matched healthy controls is summarized in **Table S1**. ALL cases were collected from Guangzhou Women and Children’s Medical Center (GWCMC), Guangzhou Medical University (n=582), The First Affiliated Hospital, Sun Yat-sen University (n=74), Sun Yat-sen Memorial Hospital, Sun Yat-sen University (n=26), Nanfang Hospital, Southern Medical University (SMU) (n=100), and Zhujiang Hospital, Southern Medical University (n=26), from January 2017 to May 2019. All children were diagnosed with ALL by at least two hematologists. The control subjects were free from any type of hematological diseases or any other malignancy or autoimmune disorder and were recruited from the same hospital.

The major clinical and biological characteristics of the ALL children, including age, gender, immunophenotype, gene fusion type, risk level, karyotype, clinical manifestations, rate of primitive/naive lymphocytes in the marrow, and minimal residual disease on 19 days, 33 days, and 12 weeks after chemotherapy and chemotherapy regimen were collected. The information is summarized in **Table S1**.

The study was approved by the institutional ethics committee of every participating hospital, and written informed consent was acquired from all participants in accordance with the Declaration of Helsinki.

### METTL3 SNPs Selection and Genotyping

The included potentially functional candidate SNPs were selected as follows: located in the 5’ untranslated region, 3’ untranslated region, 5’ flanking region, and exon of the *METTL3* gene. The NCBI dbSNP database (http://www.ncbi.nlm.nih.gov/projects/SNP) and the SNPinfo (https://snpinfo.niehs.nih.gov/snpinfo/snpfunc.html) online software were used to perform the above selection. Three SNPs (rs1263801 C>G, rs1139130 G>A, and rs1061027 A>C) in the *METTL3* gene were chosen. Genomic DNA was extracted from peripheral blood. The reaction system and condition of the Taqman RT-PCR assay was according to the published reference (20, 21). To ensure the accuracy of these genotyping results, 10% of the samples were randomly selected to be genotyped by a DNA sequencing method. A concordance rate of 100% for the quality control samples was obtained (21).

### Statistical Analysis

The goodness-of-fit χ^2^ test was performed to assess if the selected *METTL3* SNPs deviated from Hardy–Weinberg equilibrium among controls. The two-sided χ^2^ test was used to compare demographic variables and genotype frequencies of the cases and controls. ORs and their corresponding 95% CIs were computed by unconditional logistic regression analyses with or without adjustment for age and gender. The SAS statistical package (version 9.1; SAS Institute, Cary, NC) was used to perform all statistical analyses. All reported p values were two sided, and a p value < 0.05 was considered statistically significant.

## Results

### Population Characteristics

The demographic and clinical characteristics data of ALL cases and cancer-free controls are summarized in **Table S1**. No significant differences were observed between cases and controls for the Southern Chinese children regarding age (p=0.082) and gender (p=0.059). Among ALL cases, 28.22% (228 cases) were pro B cell ALL, 35.27% (285 cases) were common B cell ALL, 20.67% (167 cases) were pre-B cell ALL, 0.67% (3 cases) were mature B ALL, 8.54% (69 cases) were T cell ALL, and 6.93% (56 cases) were undefined immunophenotype. Regarding the gene fusion type, 3.34% (27 cases) had BCR-ABL gene fusion, 16.83% (136 cases) had TEL-AML, 2.97% (24 cases) had E2A-PBX, 0.99% (8 cases) had SIL-TAL, 1.98% (16 cases) had MLL, 3.09% (25 cases) had other gene fusions, 68.19% (551 cases) were normal, and 21 were undefined. A total of 258 patients (33.73%) were with low risk, 360 cases (47.06%) were with medium risk, 77 cases (10.07%) were with high risk, and 70 cases (9.15%) were undefined. Regarding the karyotype, 64.40% (517 cases) were normal diploid, 5.25% (45 cases) were abnormal diploid, 2.69% (22 cases) were hypodiploid, 3.46% (27 cases) were low hyperdiploid, and 7.81% (61 cases) were high hyperdiploid.

### Correlation of *METTL3* Gene Polymorphisms With ALL Risk

The genotype frequencies of *METTL3* associated with ALL risk are shown in **Table 1**. In the single-locus analysis, carriers of rs1263801 (CC *vs*. GG: adjusted OR= 4.18, 95% CI=3.21–5.43, p<0.001) and rs1061027 (CA *vs*. CC: adjusted OR=2.42, 95% CI=2.00–2.94, p<0.001; AA *vs*. CC: adjusted OR=6.21, 95% CI=4.38–8.81, p<0.001) variant alleles showed significant enhanced risk of pediatric ALL. On the contrary, rs1139130 (GA *vs*. GG: adjusted OR=1.41, 95% CI=1.15–1.73, p=0.001; AA *vs*. GG: adjusted OR=1.52, 95% CI=1.81–3.06, p<0.001) variant alleles contribute to decreased risk of pediatric ALL.

**Table 1:** Logistic regression analysis of associations between *METTL3* polymorphisms and ALL susceptibility.

Genotype	Cases (N = 808)	Controls (N = 1340)	P^a^	Crude OR (95% CI)	P	Adjusted OR (95% CI) b	P^b^
rs1263801 (HWE=0.0971)							
GG	269 (33.50)	600 (44.88)		1.00		1.00	
GC	304 (37.86)	611 (45.70)		1.11 (0.91-1.35)	0.305	1.12 (0.92-1.37)	0.254
CC	230 (28.64)	126 (9.42)		4.07 (3.14-5.28)	0.001	4.18 (3.21-5.43)	0.001
Additive			0.001	1.82 (1.61-2.07)	0.001	1.84 (1.63-2.09)	0.001
Dominant	534 (66.50)	737 (55.12)	0.001	1.62 (1.35-1.94)	0.001	1.64 (1.37-1.97)	0.001
Recessive	573 (71.36)	1211 (90.58)	0.001	3.86 (3.04-4.90)	0.001	3.93 (3.09-5.00)	0.001
rs1139130 (HWE=0.3401)							
GG	220 (28.17)	511 (38.51)		1.00		1.00	
GA	383 (49.04)	638 (48.08)		1.39 (1.14-1.71)	0.002	1.41 (1.15-1.73)	0.001
AA	178 (22.79)	178 (13.41)		2.32 (1.79-3.02)	0.001	2.36 (1.81-3.06)	0.001
Additive			0.001	1.51 (1.32-1.71)	0.001	1.52 (1.33-1.73)	0.001
Dominant	561 (71.83)	816 (61.49)	0.001	1.60 (1.32-1.93)	0.001	1.62 (1.34-1.96)	0.001
Recessive	603 (77.21)	1149 (86.59)	0.001	1.90 (1.51-2.40)	0.001	1.92 (1.52-2.42)	0.001
rs1061027 (HWE=0.6433)							
CC	319 (39.78)	859 (64.73)		1.00		1.00	
CA	364 (45.39)	414 (31.20)		2.37 (1.96-2.87)	0.001	2.42 (2.00-2.94)	0.001
AA	119 (14.84)	54(4.07)		5.93 (4.20-8.39)	0.001	6.21 (4.38-8.81)	0.001
Additive			0.001	2.41 (2.09-2.78)	0.001	2.46 (2.13-2.84)	0.001
Dominant	483 (60.33)	468 (35.27)	0.001	2.78 (2.32-3.33)	0.001	2.85 (2.38-3.42)	0.001
Recessive	683 (85.16)	1273 (95.93)	0.001	4.11 (2.94-5.74)	0.001	4.23 (3.02-5.93)	0.001

a *χ*^2^ test for genotype distributions between ALL cases and cancer-free controls.

b Adjusted for age and gender.

### Stratification Analysis of Identified SNPs

We further analyzed whether the selected *METTL3* polymorphisms (rs1263801 C>G, rs1139130 A>G, and rs1061027 A>C) preferentially predispose to any specific subtype of ALL (**Table 2**). A stronger risk effect of rs1263801 was found among children older than 10 years (adjusted OR= 4.65, 95% CI=2.39–9.05, p<0.001), male (adjusted OR= 4.06, 95% CI=2.97–5.54, p=0.001), common B subtype ALL (adjusted OR= 8.59, 95% CI=6.37–11.6, p<0.001), MLL gene fusion type (adjusted OR= 13.1, 95% CI=4.77–35.9, p<0.001), low hyperdiploid (adjusted OR= 6.16, 95% CI=2.78–13.7, p<0.001), primitive/naive lymphocytes in marrow ≥ 5% on 19 days (adjusted OR=4.83, 95% CI=2.86–8.24, p<0.001) after chemotherapy, primitive/naive lymphocytes in marrow < 5% on 33 days (adjusted OR= 3.90, 95% CI=2.98–5.10, p<0.001) and 12 weeks (adjusted OR=1.65, 95% CI=1.15–2.36, p=0.007) after chemotherapy, MRD in marrow < 0.01% on 19 days (adjusted OR=10.6, 95% CI=5.81–19.5, p<0.001), MRD ≥ 0.01% on 33 days (adjusted OR=5.64, 95% CI=4.07–7.80–8.24, p<0.001), and ≥0.01% on 12 weeks (adjusted OR=2.89, 95% CI=1.14–7.35, p=0.026). As for the rs1139130 polymorphism, a more significant risk association was identified with those children age ≥10 years (adjusted OR=2.07, 95% CI= 1.06–4.05, p=0.034), female (adjusted OR=2.62, 95% CI= 18.1–3.78, p<0.001), common B subtype (adjusted OR=3.58, 95% CI= 2.67–4.81, p<0.001), MLL gene fusion type (adjusted OR=3.95, 95% CI=1.41–11.0, p=0.009), normal diploid (adjusted OR=1.88, 95% CI=1.45–2.45, p<0.001), primitive/naive lymphocytes in marrow ≥ 5% on 19 days (adjusted OR= 2.27, 95% CI=1.29–4.00, p<0.001) and <5% on 33 days (adjusted OR=1.72, 95% CI=1.32–2.26, p<0.001) after chemotherapy, MRD in marrow <0.01% on 19 days (adjusted OR= 4.66, 95% CI=2.52–8.60, p<0.001), MRD ≥ 0.01% on 33 days (adjusted OR= 2.19, 95% CI=1.56–3.06, p<0.001), and ≥0.01% on 12 weeks (adjusted OR= 2.91, 95% CI=1.24–6.81, p=0.014). As for the rs1061027 polymorphism, a stronger risk association was revealed with those children age ≥10 years (adjusted OR=7.10, 95% CI= 2.78–18.1, p<0.001), female (adjusted OR=4.84, 95% CI= 2.83–8.28, p<0.001), common B subtype (adjusted OR=9.72, 95% CI= 6.67–14.2, p<0.001), MLL gene fusion type (adjusted OR=14.8, 95% CI= 5.15–42.6, p<0.001), low hyperdiploid (adjusted OR=7.83, 95% CI= 3.17–19.5, p<0.001), primitive/naive lymphocytes in marrow ≥5% on 19 days (adjusted OR= 5.69, 95% CI=2.96–10.9, p<0.001) and <5% on 33 days (adjusted OR=2.72, 95% CI= 1.82–4.05, p<0.001) after chemotherapy, MRD in marrow <0.01% on 19 days (adjusted OR= 13.1, 95% CI=6.80–25.3, p<0.001), and MRD ≥0.01% on 33 days (adjusted OR= 3.02, 95% CI=1.84–4.92, p<0.001). No correlation was found between the rs1061027 polymorphism and MRD on 12 weeks.

**Table 2:** Stratification analysis of *METTL3* polymorphisms with ALL susceptibility.

Variables	rs1263801 (cases/controls)		Adjusted OR^a^	*P* ^a^	rs1139130 (cases/controls)		Adjusted OR^a^	*P* ^a^	rs1061027 (cases/controls)		Adjusted OR^a^	*P* ^a^
	GG/GC	CC	(95% CI)		GG/GA	AA	(95% CI)		CC/CA	AA	(95% CI)	
Age, month												
<120	513/1095	192/110	3.69 (2.85-4.77)	0.001	534/1036	154/159	1.87 (1.46-2.40)	0.001	610/1149	94/48	3.64 (2.53-5.22)	0.001
≥120	60/116	38/16	4.65 (2.39-9.05)	0.001	69/113	24/19	2.07 (1.06-4.05)	0.034	73/124	25/6	7.10 (2.78-18.1)	0.001
Gender												
Females	227/435	96/50	3.72 (2.55-5.44)	0.001	226/426	85/60	2.62 (1.81-3.78)	0.001	268/462	53/20	4.84 (2.83-8.28)	0.001
Males	346/776	134/76	4.06 (2.97-5.54)	0.001	377/723	93/118	1.53 (1.13-2.06)	0.005	415/811	64/34	3.82 (2.47-5.92)	0.001
Immunophenotyping												
Pro B	198/1211	29/126	1.47 (0.95-2.28)	0.083	181/1149	41/178	1.51 (1.04-2.21)	0.032	214/1273	13/54	1.51 (0.80-2.83)	0.201
Common B	149/1211	133/126	8.59 (6.37-11.6)	0.001	177/1149	98/178	3.58 (2.67-4.81)	0.001	199/1273	82/54	9.72 (6.67-14.2)	0.001
Pre B	136/1211	30/126	2.22 (1.43-3.44)	0.001	140/1149	22/178	1.04 (0.65-1.68)	0.863	158/1273	8/54	1.25 (0.58-2.68)	0.570
Mature B	3/1211	0/126	0.001 (0.00-999)	0.973	3/1149	0/178	0.001 (0.00-999)	0.968	3/1273	0/54	0.001(0.00-999)	0.982
T ALL	49/1211	20/126	3.78 (2.15-6.63)	0.001	58/1149	8/178	0.86 (0.40-1.83)	0.688	61/1273	8/54	2.88 (1.29-6.42)	0.010
Mix	38/1211	18/126	4.41 (2.43-7.99)	0.001	44/1149	9/178	1.31 (0.63-2.73)	0.474	48/1273	8/54	3.82 (1.71-8.53)	0.002
Gene fusion type												
BCR-ABL	15/1211	12/126	6.05 (2.66-13.7)	0.001	20/1149	6/178	1.63 (0.62-4.26)	0.319	20/1273	7/54	6.15 (2.32-16.3)	0.001
TEL-AML	96/1211	39/126	4.08 (2.68-6.21)	0.001	103/1149	30/178	1.89 (1.22-2.93)	0.004	114/1273	21/54	4.61 (2.67-7.94)	0.001
E2A-PBX	21/1211	3/126	1.40 (0.41-4.79)	0.588	21/1149	2/178	0.65 (0.15-2.80)	0.562	23/1273	1/54	1.05 (0.14-7.93)	0.965
SIL-TAL	7/1211	1/126	1.31 (0.16-10.9)	0.797	7/1149	0/178	0.001 (0.00-999)	0.961	7/1273	1/54	3.27 (0.39-27.2)	0.274
MLL	7/1211	9/126	13.1 (4.77-35.9)	0.001	10/1149	6/178	3.95 (1.41-11.0)	0.009	10/1273	6/54	14.8 (5.15-42.6)	0.001
Others	10/1211	15/126	14.2 (6.22-32.3)	0.001	13/1149	11/178	5.53 (2.44-12.6)	0.001	16/1273	9/54	13.2 (5.56-31.3)	0.001
Normal	405/1211	142/126	3.41 (2.61-4.45)	0.001	413/1149	118/178	1.86 (1.43-2.41)	0.001	474/1273	72/54	3.63 (2.50-5.25)	0.001
Karyotype												
Hypo-diploid	17/1211	5/126	2.83 (1.02-7.85)	0.046	15/1149	5/178	1.84 (0.67-5.07)	0.236	20/1273	2/54	2.29 (0.52-10.1)	0.274
Normal diploid	371/1211	144/126	3.78 (2.89-4.95)	0.001	389/1149	113/178	1.88 (1.45-2.45)	0.001	434/1273	80/54	4.44 (3.08-6.39)	0.001
Abnormal diploid	34/1211	11/126	2.99 (1.47-6.06)	0.002	35/1149	7/178	1.28 (0.56-2.93)	0.562	40/1273	5/54	2.87 (1.09-7.58)	0.034
Low hyperdiploid	16/1211	11/126	6.16 (2.78-13.7)	0.001	20/1149	5/178	1.58 (0.58-4.27)	0.372	20/1273	7/54	7.83 (3.17-19.5)	0.001
High hyperdiploid	45/1211	16/126	3.66 (1.99-6.71)	0.001	49/1149	12/178	1.67 (0.87-3.22)	0.126	57/1273	4/54	1.81 (0.63-5.22)	0.271
Primitive/naive lymphocytes in marrow(%, 19d)												
<5	362/1211	134/126	3.66 (2.79-4.82)	0.001	377/1149	104/178	1.80 (1.38-2.36)	0.001	441/1273	55/54	3.07 (2.07-4.56)	0.001
≥5	45/1211	24/126	4.83 (2.86-8.24)	0.001	50/1149	18/178	2.27 (1.29-4.00)	0.004	55/1273	14/54	5.69 (2.96-10.9)	0.001
MRD in marrow(%, 19d)												
<0.01	22/1211	25/126	10.6 (5.81-19.5)	0.001	26/1149	19/178	4.66 (2.52-8.60)	0.001	30/1273	17/54	13.1 (6.80-25.3)	0.001
≥0.01	298/1211	159/126	5.27 (4.03-6.89)	0.001	337/1149	108/178	2.09 (1.60-2.73)	0.001	378/1273	79/54	5.18 (3.58-7.49)	0.001
Primitive/naïve lymphocytes in marrow(%, 33d)												
<5	367/1211	144/126	3.90 (2.98-5.10)	0.001	396/1149	104/178	1.72 (1.32-2.26)	0.001	460/1273	51/54	2.72 (1.82-4.05)	0.001
≥5	27/1211	5/126	1.83 (0.69-4.87)	0.223	23/1149	6/178	1.80 (0.72-4.50)	0.211	30/1273	2/54	1.61 (0.37-6.95)	0.523
MRD in marrow(%, 33d)												
<0.01	242/1211	50/126	2.06 (1.44-2.95)	0.001	231/1149	48/178	1.36 (0.96-1.94)	0.082	262/1273	30/54	2.83 (1.77-4.52)	0.001
≥0.01	149/1211	86/126	5.64 (4.07-7.80)	0.001	175/1149	59/178	2.19 (1.56-3.06)	0.001	209/1273	26/54	3.02 (1.84-4.92)	0.001
Primitive/naïve lymphocytes in marrow(%, 12w)												
<5	288/1211	47/126	1.65 (1.15-2.36)	0.007	269/1149	52/178	1.29 (0.92-1.81)	0.139	312/1273	23/54	1.90 (1.14-3.16)	0.014
≥5	12/1211	1/126	0.88 (0.11-6.92)	0.907	10/1149	3/178	2.14 (0.58-7.96)	0.257	13/1273	0/54	0.001 (0.00-999)	0.980
MRD in marrow(%, 12w)												
<0.01	282/1211	36/126	1.31 (0.88-1.94)	0.187	262/1149	43/178	1.10 (0.77-1.58)	0.600	299/1273	19/54	1.67 (0.97-2.88)	0.066
≥0.01	20/1211	6/126	2.89 (1.14-7.35)	0.026	18/1149	8/178	2.91 (1.24-6.81)	0.014	25/1273	1/54	0.95 (0.13-7.16)	0.962

a Adjusted for age and gender.

### Association of *METTL3* Polymorphisms With Chemotherapeutics in Southern Chinese Pediatric ALL Patients

All patients were treated with Chinese Children Cancer Group chemotherapeutics (CCCG) or South China Children Leukemia Group chemotherapeutics (SCCLG). We compared the MRD in the marrow of patients with different alleles after being treated with CCCG and SCCLG (**Table 3**). As for rs1263801, CC alleles provided a protective effect on MRD in the marrow ≥0.01% on 33 days (adjusted OR= 0.17, 95% CI= 0.10–0.30, p<0.001) and 12 weeks (adjusted OR= 0.30, 95% CI= 0.10–0.90, p=0.030) among CCCG-treated children. As for rs1139130, AA alleles provided a protective effect on MRD in marrow ≥0.01% on 33 days (adjusted OR= 0.50, 95% CI= 0.29–0.83, p=0.008) and 12 weeks (adjusted OR= 0.32, 95% CI= 0.12–0.87, p=0.030) among CCCG-treated children but provided a risk effect on MRD in marrow ≥0.01% among SCCLG-treated children (adjusted OR=5.70, 95% CI=1.37–23.7, p=0.017). As for rs1061.27, AA alleles provided a risk effect on MRD in the marrow ≥0.01% among CCCG-treated children (adjusted OR=8.63, 95% CI=2.31–32.3, p=0.002). These results indicated that SCCLG chemotherapeutics is more suitable for rs1263801 CC and rs1139130 AA carriers; CCCG chemotherapeutics is more efficient for rs1061027 AA carriers.

**Table 3:** The correlation between METTL3 polymorphisms and South China pediatric ALL patients’ response to different chemotherapeutics.

SNP	Variables		MRD in marrow (%, 19d)				MRD in marrow (%, 33d)				MRD in marrow (%, 12w)			
			Case (%)		*P* ^a^	Adjusted OR^a^	Case (%)		*P* ^a^	Adjusted OR^a^	Case (%)		*P* ^a^	Adjusted OR^a^
			<0.01	≥0.01		(95% CI)	<0.01	≥0.01		(95% CI)	<0.01	≥0.01		(95% CI)
rs1263801	CCCG-ALL-2015	GG/GC	4 (1.54)	256 (98.46)			194 (59.88)	130 (40.12)			238 (93.70)	16 (6.30)		
		CC	6 (4.69)	122 (95.31)	0.09	3.00 (0.83-10.9)	19 (20.88)	72 (79.12)	0	0.17 (0.10-0.30)	21 (80.77)	5 (19.23)	0.03	0.30 (0.10-0.90)
													
	SCCLG-ALL-2016	GG/GC	7 (23.33)	23 (76.67)			25 (71.43)	10 (28.57)			26 (89.66)	3 (10.34)		
		CC	9 (34.62)	17 (65.38)	0.31	1.89 (0.55-6.48)	19 (73.08)	7 (26.92)	0.6	1.39 (0.41-4.72)	9 (90.00)	1 (10.00)	0.64	1.85 (0.14-25.4)
rs1139130	CCCG-ALL-2015	GG/GA	4 (1.41)	279 (98.59)			173 (53.07)	153 (46.93)			215 (93.89)	14 (6.11)		
		AA	4 (4.40)	87 (95.60)	0.14	2.88 (0.70-11.9)	27 (36.00)	48 (64.00)	0.01	0.50 (0.29-0.83)	31 (81.58)	7 (33.33)	0.03	0.32 (0.12-0.87)
													
	SCCLG-ALL-2016	GG/GA	8 (20.00)	32 (80.00)			32 (71.11)	13 (28.89)			28 (89.66)	7 (10.34)		
		AA	8 (50.00)	8 (50.00)	0.02	5.70 (1.37-23.7)	12 (75.00)	4 (25.00)	0.53	1.56 (0.39-6.31)	7 (100.0)	0 (00.00)	0.97	999 (0.00-999)
rs1061027	CCCG-ALL-2015	CC/CA	4 (1.22)	324 (98.78)			205 (52.03)	189 (47.97)			251 (92.28)	21 (7.72)		
		AA	6 (10.00)	54 (90.00)	0	8.63 (2.31-32.3)	8 (38.10)	13 (61.90)	0.19	0.54 (0.22-1.35)	8 (100.0)	0 (0.00)	0.98	999 (0.00-999)
													
	SCCLG-ALL-2016	CC/CA	9 (25.71)	26 (74.29)			31 (75.61)	10 (24.39)			28 (90.32)	3 (9.68)		
		AA	7 (33.33)	14 (66.67)	0.4	1.74 (0.47-6.37)	13 (65.00)	7 (35.00)	0.61	0.72 (0.21-2.51)	7 (87.50)	1 (12.50)	0.77	1.50 (0.10-21.8)

^a^Adjusted for age and gender.

CCCG, Chinese Children Cancer Group; SCCLG, South China Children Leukemia Group.

## Discussion

In the current case-control study with 808 pediatric ALL case and 1,340 healthy controls from Southern Chinese populations, we explored the potential association between *METTL3* gene polymorphisms and pediatric ALL risk. We certificated that three polymorphisms, namely rs1263801 C>G, rs1139130 A>G, and rs1061027 A>C, were associated with an increased susceptibility of pediatric ALL. To our knowledge, this study is the first to identify the association between *METTL3* polymorphisms and pediatric ALL susceptibility.

Epigenetic alterations, including DNA methylation, histone modifications, and noncoding RNAs, have been reported to contribute to ALL progression (22). In recent years, another epigenetic modification, RNA methylation, is considered to play an important role in carcinogenesis (11). M6A is the most common modification of RNA on the posttranscriptional level, mainly in mRNA and long noncoding RNA (lncRNA) (23). The complex composed of METTL3, METTL14, and WTAP induces m6A-methylation of mRNA or lncRNA. METTL3 is the essential component of the complex. Dysregulation of METTL3 was identified to be a key role in the progression of multiple malignant tumors, such as endometrial cancer (24), bladder cancer (25), pancreatic cancer (26), etc. A number of articles infer that METTL3 can promote tumor progression through multiple mechanisms. METTL3 can promote growth, survival, and invasion by interacting with the translation initiation element to enhance mRNA translation in lung adenocarcinoma (27). Lin et al. (28) revealed that deletion of METTL3 could impair the epithelial-mesenchymal transition (EMT). In breast cancer, METTL3 is upregulated by HBXIP and promotes the cancer progression by suppressing let-7g (29). METTL3 promotes self-renewal of glioblastoma stem cells to induce tumorigenesis (30). METTL3 can directly interact with the eukaryotic translation initiation factor e subunit h (eIF3h). The interaction between METTL3 and eIF3h is essential for translation and oncogenic transformation in lung cancer (31). Promoter-bound METTL3 promotes m6A modification within the coding region of mRNA transcript and enhances translation by inhibiting ribosome stalling. METTL3 regulates mRNA expression in this way to facilitate the progression of acute myeloid leukemia (32). However, the function of METTL3 in ALL is still unknown.

Herein, we investigated whether *METTL3* gene polymorphisms could influence the susceptibility of ALL in South China children for the first time. With regard to the remaining three *METTL3* gene polymorphisms (rs1263801 C>G, rs1139130 A>G, and rs1061027 A>C), we identified the association between these three SNPs and pediatric ALL susceptibility. The location and predicted function was analyzed using the online software SNPinfo. The rs1263801 C>G polymorphism was located in intron 1 of the *METTL3* gene and was predicted to be the transcriptional factor binding site. The rs1139130 A>G located in the exon 5 of the *METTL3* gene was predicted to affect splicing and protein function. The rs1061027 A>C polymorphism located in intron 8 was predicted to be associated with miRNA function. In 2018, Bertero et al. reported that the interactome of transcriptional factors SMAD2/3 promoted another transcriptional factor TGFβ to control the METTL3/METTL14/WTAP complex mediated m6A mRNA methylation in human pluripotent stem cells (33). Xia et al. reported that *Zmettl3* mutation disrupts gamete maturation and reduces fertility in zebrafish (34). Other studies identified that METTL3 mRNA could be targeted by miR-600 (35) and miR-33a (36). However, there was no evidence certifying that genetic variations of METTL3 could affect the transcriptional factor or miRNAs binding to METLL3 and the coding of METTL3 mRNA. Our results suggested that the rs1263801 CC phenotype, rs1139130 GG phenotypes, and rs1061027 CA/CC phenotypes are associated with an increased risk of pediatric ALL in South China. Lin et al. reported that the combination of rs1139130, rs1263801, rs1061026, and rs1061027 reduced the risk of Wilms tumor in Chinese children (37). Bian et al. (38) identified that these four polymorphisms were associated with an increased risk of neuroblastoma. It suggested that *METTL3* polymorphisms function diversely in different tumors.

We next examined whether the *METTL3* SNP genotype preferentially predisposes to any pediatric ALL subtype, including immunophenotype, gene fusion type, karyotype, primitive/naïve lymphocytes, and MRD in the marrow after chemotherapy. The *METTL3* rs1263801 CC phenotype and the rs1061027 AA phenotype were considered to increase the risk of ALL in the B-ALL, mature B ALL, and T-ALL subtype. In BCR-ABL, TEL-AML, and MLL gene fusion types, rs1263801 CC phenotype and rs1061027 AA phenotype carriers showed a higher risk for ALL. The rs1139130 GG carriers were revealed to have a higher risk for ALL in B-ALL, mature B ALL subtype, and medium risk level subtype. We failed to identify the association between the FAB subtype and these three *METTL3* polymorphisms.

In stratification analysis, we tried to reveal the relationship between clinical characteristic, response to different chemotherapeutics, and *METTL3* polymorphisms. The results showed that rs1263801 C>G, rs1139130 A>G, and rs1061027 A>C could remarkably increase the risk of the common B type and MLL fusion type ALL in Southern Chinese children. All these three selected polymorphisms were more strongly associated with the primitive/naïve lymphocytes over 5% and MRD less than 0.01% on the 19th day, and also with the primitive/naïve lymphocytes less than 5% and MRD more than 0.01% on the 33rd day after chemotherapy. After chemotherapy treatment of 12 weeks, rs1263801 C>G and rs1061027 A>C were identified to increase susceptibility to primitive/naïve lymphocytes less than 5%; rs1263801 C>G and rs1139130 A>G may increase susceptibility to MRD more than 0.01% in ALL patients. And we also identified that SCCLG chemotherapeutics was more suitable for rs1263801 CC and rs1139130 AA carriers; CCCG chemotherapeutics was more efficient for rs1061027 AA carriers.

Several limitations should be noted in the current study. First, the sample size was not large enough. Second, this was a retrospective study; information bias and selection bias were inevitable. We have reduced these biases by frequency-matching of cases and controls by age and gender, and recruiting subjects from six hospitals in South China. Third, our study focused on the analysis of genetic factors in pediatric ALL risk. However, other important information such as environment and dietary intake was not available for analysis. Finally, the association between *METTL3* gene polymorphisms and prognosis of pediatric ALL was not analyzed in the current study.

In summary, our results suggest that polymorphisms rs1263801 C>G, rs1139130 A>G, and rs1061027 A>C in the *METTL3* gene were significantly associated with increased pediatric ALL risk, and SCCLG chemotherapeutics is more suitable for rs1263801 CC and rs1139130 AA carriers; CCCG chemotherapeutics is more efficient for rs1061027 AA carriers in the Southern Chinese ALL children. Further studies are necessary to elucidate the biological function of *METTL3* gene risk SNPs in the etiology of pediatric ALL.

## Conclusion

*METTL3* gene polymorphisms were associated with increased pediatric ALL risk. These three polymorphisms (rs1263801 C>G, rs1139130 A>G, and rs1061027 A>C) were likely to contribute to the sensitivity of different chemotherapies in pediatric ALL. The results indicated that METTL3 gene polymorphisms might be a potential biomarker for ALL susceptibility and when choosing chemotherapeutics.

## Data Availability Statement

The datasets presented in this study can be found in online repositories. The names of the repository/repositories and accession number(s) can be found in the article/**Supplementary Material**.

## Ethics Statement

The study was approved by the institutional ethics committee of Guangzhou Women and Children’s Medical Center, Guangzhou Medical University; The First Affiliated Hospital, Sun Yat-sen University; Sun Yat-sen Memorial Hospital, Sun Yat-sen University; Nanfang Hospital, Southern Medical University; and Zhujiang Hospital, Southern Medical University. Written informed consent to participate in this study was provided by the participants’ legal guardian/next of kin.

## Author Contributions

XpL and LH are equal to this work in writing the manuscript. KH, LY, XW, JW, and YC collected ALL blood samples. XY analyzed the data. AL, MC, XdL, and YY performed qPCR. LX and HJ supplied the idea and funding. All authors contributed to the article and approved the submitted version.

## Funding

National Natural Science Foundation of China (81672496 and 81870115), Natural Science Foundation of Guangdong Province (2020A1515010188), and Guangzhou Municipal Science and Technology Project (201804010042).

## Conflict of Interest

The authors declare that the research was conducted in the absence of any commercial or financial relationships that could be construed as a potential conflict of interest.

## Publisher’s Note

All claims expressed in this article are solely those of the authors and do not necessarily represent those of their affiliated organizations, or those of the publisher, the editors and the reviewers. Any product that may be evaluated in this article, or claim that may be made by its manufacturer, is not guaranteed or endorsed by the publisher.
