# Supplementary material for: Novel Associations Between METTL3 Gene Polymorphisms and Pediatric Acute Lymphoblastic Leukemia: A Five-Center Case-Control Study
Source: Front Oncol. 2021 Sep 9;11:635251. doi: 10.3389/fonc.2021.635251 (PMC8459019; doi:10.3389/fonc.2021.635251)
Supplement: Supplementary file 1 [file Table_1.docx]

| **Table S1**. Frequency distribution of selected characteristics in ALL cases and cancer-free controls | | | | | | |
| --- | --- | --- | --- | --- | --- | --- |
| Variables | ALL | | | | | |
|  | Cases (n=808) | | | Controls (n=1340) | |  |
|  | No. | % | | No. | % |  |
| Age range, year | 0.50-17 | | | 0.91-15 | |  |
| Mean ± SD | 5.14±3.15 | | | 5.39±2.76 | |  |
| <120 | 709 | | 87.75 | 1208 | 90.15 |  |
| ≥120 | 99 | | 12.25 | 132 | 9.85 |  |
| Gender |  | |  |  |  |  |
| Female | 326 | | 40.35 | 486 | 36.27 |  |
| Male | 482 | | 59.65 | 854 | 63.73 |  |
| Hospital |  | |  |  |  |  |
| GWCMC | 582 | | 72.03 | 966 | 72.09 |  |
| Nanfang Hospital, SMU | 100 | | 12.38 | 165 | 12.31 |  |
| The First Affiliated Hospital, SYSU | 74 | | 9.16 | 123 | 9.18 |  |
| Zhujiang Hospital, SMU | 26 | | 3.22 | 43 | 3.21 |  |
| Sun Yat-sen Memorial Hospital, SYSU | 26 | | 3.22 | 43 | 3.21 |  |
| Immunophenotyping |  | |  |  |  |  |
| Pro B ALL | 228 | | 28.22 |  |  |  |
| Common B ALL | 285 | | 35.27 |  |  |  |
| Pre B ALL | 167 | | 20.67 |  |  |  |
| Mature B ALL | 3 | | 0.37 |  |  |  |
| T ALL | 69 | | 8.54 |  |  |  |
| NA | 56 | | 6.93 |  |  |  |
| Gene fusion type |  | |  |  |  |  |
| BCR-ABL | 27 | | 3.34 |  |  |  |
| TEL-AML | 136 | | 16.83 |  |  |  |
| E2A-PBX | 24 | | 2.97 |  |  |  |
| SIL-TAL | 8 | | 0.99 |  |  |  |
| MLL | 16 | | 1.98 |  |  |  |
| Others | 25 | | 3.09 |  |  |  |
| Normal | 551 | | 68.19 |  |  |  |
| NA | 21 | | 2.60 |  |  |  |
| Risk level |  | |  |  |  |  |
| Low | 258 | | 33.73 |  |  |  |
| Medium | 360 | | 47.06 |  |  |  |
| High | 77 | | 10.07 |  |  |  |
| NA | 70 | | 9.15 |  |  |  |
| Karyotype |  | |  |  |  |  |
| Normal diploid | 517 | | 64.40 |  |  |  |
| Abnormal diploid | 45 | | 5.25 |  |  |  |
| Hypo-diploid | 22 | | 2.69 |  |  |  |
| Low hyperdiploid | 27 | | 3.46 |  |  |  |
| High hyperdiploid | 61 | | 7.81 |  |  |  |
| NA | 136 | | 16.39 |  |  |  |
| SD, standard deviation; NA, not available.  ^a^ Two-sided *χ^2^* test for distributions between ALL cases and cancer-free controls. | | | | | | |
